# Are Multicentre Bond Indices and Related Quantities Reliable Predictors of Excited-State Aromaticity?

**DOI:** 10.3390/molecules25204791

**Published:** 2020-10-19

**Authors:** Robert Ponec, David L. Cooper, Peter B. Karadakov

**Affiliations:** 1 Institute of Chemical Process Fundamentals, Czech Academy of Sciences, 165 02 Prague 6 Suchdol 2, Czech Republic; 2 Department of Chemistry, University of Liverpool, Liverpool L69 7ZD, UK; dlc@liverpool.ac.uk; 3 Department of Chemistry, University of York, Heslington, York YO10 5DD, UK; peter.karadakov@york.ac.uk

**Keywords:** excited-state aromaticity reversals, multicentre bond indices, molecular similarity, magnetic properties of excited states

## Abstract

Systematic scrutiny is carried out of the ability of multicentre bond indices and the NOEL-based similarity index *d_AB_* to serve as excited-state aromaticity criteria. These indices were calculated using state-optimized complete active-space self-consistent field wavefunctions for several low-lying singlet and triplet states of the paradigmatic molecules of benzene and square cyclobutadiene and the inorganic ring S_2_N_2_. The comparison of the excited-state indices with aromaticity trends for individual excited states suggested by the values of magnetic aromaticity criteria show that whereas the indices work well for aromaticity reversals between the ground singlet and first triplet electronic states, addressed by Baird’s rule, there are no straightforward parallels between the two sets of data for singlet excited states. The problems experienced while applying multicentre bond indices and *d_AB_* to singlet excited states are explained by the loss of the information inherently present in wavefunctions and/or pair densities when calculating the first-order density matrix.

## 1. Introduction

Despite its somewhat vaguely defined qualitative nature, the concept of aromaticity has had huge impacts on organic chemistry, starting with the formulation of the Hückel aromaticity rules [1,2] and encompassing a broad research area including the elucidation of the link between cyclic delocalization and energetic stabilization of conjugated (poly)cyclic hydrocarbons [3,4,5,6,7,8,9,10], the role of cyclic conjugation in inducing the ring currents [11,12,13,14,15,16,17,18,19] responsible for the special magnetic properties of aromatic compounds, and revealing the links between electron counts, orbital topology and selection rules in pericyclic reactions [20,21,22,23]. The fact that the phenomenon of aromaticity can be associated with a very wide range of structural, energetic, and magnetic properties [3,4,5,6,7,8,9,10,11,12,13,14,16,17,18,24,25] has given impetus to numerous attempts to define measures or indices that are intended to characterize the “extent” of aromaticity in quantitative terms [16,17,18,19,24,25,26,27,28,29,30,31,32,33]. However, such efforts have often been plagued by discrepancies between the various types of indices; these have led to the postulation of a multidimensional character for this phenomenon [34,35,36], even implying “orthogonality” between energetic and magnetic measures of aromaticity as manifested by the reported absence of a straightforward link between these two types of aromaticity measure [19,29,37,38,39]. However, it has been demonstrated that such discrepancies are most often observed when trying to juxtapose quantities that are not straightforward to compare. One such example is provided by attempts to correlate the extents of cyclic delocalization in the individual benzene rings in polycyclic aromatic hydrocarbons (PAHs), as given by multicentre bond indices, with the values of nucleus-independent chemical shieldings (NICS) [18]. This fails not least because of the incompatibility between the strictly local character of multicentre indices and the fact that the NICS value for an individual ring is “contaminated” by the interfering contributions of the other rings [37,39]. The agreement between these two types of index is in fact restored when the contaminating contributions are properly taken into account; analogous parallels have been established between multicentre indices, induced ring currents and (when properly accounted for) the energetic effects of cyclic conjugation [19,37,38,39,40,41].

Although initially most studies were focused on aromaticity in ground electronic states, Baird’s pioneering discovery of the reversal of Hückel’s aromaticity rules upon electronic excitation from the singlet ground to the first triplet excited state [42] directed attention to the systematic investigation of excited-state aromaticity [43,44,45,46,47,48,49,50,51]. The importance of such studies for the understanding of the photochemical/physical properties of photoactive materials has prompted the development of experimental and computational tools that are capable of providing reliable estimates of excited-state aromaticity. Amongst the first attempts at theoretical justification of Baird’s discovery of aromaticity reversals in the lowest excited states of cyclic conjugated hydrocarbons is a study by Iljić et al. [43] which looked at the extension of the concept of topological resonance energy (TRE) to low-lying states of cyclic conjugated hydrocarbons. The authors of that study demonstrated that the TRE values for the ground and lowest excited states of conjugated rings reproduce the aromaticity reversal predicted by Baird’s rule. Despite the elegant simplicity of this approach, the calculation of TREs has serious inherent limitations arising from the Hückel molecular orbital (HMO) foundations of the underlying graph-theoretical considerations. Modern quantum chemical calculations are not subject to such limitations and the scope of excited-state aromaticity studies was subsequently extended to formulating Baird-style rules for higher excited states. The most convincing proof of aromaticity and/or antiaromaticity reversals in the first and higher excited states was provided by the results of systematic studies of various magnetic properties with state-specific complete active-space self-consistent field (CASSCF) wavefunctions constructed from gauge-included atomic orbitals (GIAOs) [44,45,46,52]. Given that multicentre bond indices have been applied successfully for the quantitative evaluation of the local aromaticities of individual benzene rings in PAHs [28,29,37,40], it was natural to try to find out whether the same approach could provide a computationally efficient and sufficiently accurate characterization of excited-state aromaticity. The aim of the current work is to carry out a systematic comparative study of the performance and reliability of multicentre bond indices and other first-order density-based quantities for the description and classification of excited-state aromaticity in the paradigmatic molecules benzene and cyclobutadiene, as well as in disulfur dinitride, which has been shown recently to be the first inorganic ring that exhibits changes in aromaticity between different electronic states [52]. As will be shown, it turns out that such quantities have significant difficulties distinguishing properly between singlet diradical and zwitterionic character.

## 2. Computational Methodology

### 2.1. Electronic Structure Calculations

The aromaticity of the low-lying electronic states of benzene, square cyclobutadiene and disulfur dinitride has been analysed using a range of magnetic criteria including NICS calculated with CASSCF-GIAO wavefunctions at fixed ground electronic state geometries [27,44,45,46,52]. To enable direct comparisons, we use the same levels of theory and the same geometries in the current work. All excited electronic state properties discussed in this work correspond to vertical excitations because we chose to use identical ground-state geometries for all electronic states of a given molecule. All CASSCF calculations on benzene and cyclobutadiene reported in this paper were carried out within the 6-311++G(2d,2p) basis, whereas use was made of the cc-pVTZ basis for S_2_N_2_.

It is important in this work to focus on vertical excitations not least because the electronic wavefunction changes much more rapidly than the molecular geometry. By examining the excited-state wavefunction at the ground-state geometry we can establish whether a given vertically excited state is intrinsically aromatic, antiaromatic or non-aromatic. If it turns out to be aromatic, then it will of course tend to retain a geometry that is similar to that of the ground state. If, on the other hand, it is antiaromatic then it is likely to experience a geometry distortion that leads to a lower energy, less antiaromatic and closer to non-aromatic geometry. The same does of course apply for systems which are antiaromatic in their electronic ground states, such as the ground state of square (*D*_4*h*_) cyclobutadiene; the relaxation of the geometry of that system to rectangular (*D*_2*h*_) decreases the antiaromaticity, not only making this state much more non-aromatic but also rendering it significantly less interesting to study as an example of an antiaromatic molecule.

The S_0_ (1 ^1^A_1g_), T_1_ (1 ^3^B_1u_), S_1_ (1 ^1^B_2u_) and S_2_ (1 ^1^B_1u_) electronic states of benzene were described using state-optimized π-space CASSCF(6,6) wavefunctions (with “6 electrons in 6 orbitals”). We used the experimental *D*_6*h*_ gas-phase ground-state geometry with C−C and C−H bond lengths of 1.3964 Å and 1.0831 Å, respectively, which was obtained through analysis of the ν_4_ vibration-rotation bands of C_6_H_6_ and C_6_D_6_ [53].

The calculations for the S_0_ (1 ^1^B_1g_), T_1_ (1 ^3^A_2g_), S_1_ (1 ^1^A_1g_) and S_2_ (1 ^1^B_2g_) electronic states of square (*D*_4*h*_) cyclobutadiene employed state-optimized π-space CASSCF(4,4) wavefunctions (with “4 electrons in 4 orbitals”). We used C−C and C−H bond lengths of 1.447 Å and 1.076 Å, respectively, that were optimized with the cc-pVTZ basis through a multireference averaged quadratic coupled cluster (MR-AQCC) approach, taking orbitals from corresponding state-averaged π-space CASSCF(4,4) wavefunctions that included the ground state, lowest triplet state and two lowest singlet excited states (SA-4-CASSCF) [54].

The calculations on the S_0_ (1 ^1^A_g_), T_1_ (1 ^3^B_3u_) and S_1_ (1 ^1^A_u_) electronic states of S_2_N_2_ were carried out using state-optimized CASSCF(22,16) wavefunctions (with “22 electrons in 16 orbitals”). For this purpose we used the *D*_2*h*_ semi-experimental equilibrium geometry established by Perrin et al. [55], with *R*(SN) = 1.64182 Å and ∠(NSN) = 91.0716°, in a coordinate system that places N at positions (±1.171748 Å, 0.0, 0.0), and S atoms at positions (0.0, ±1.150035 Å, 0.0), respectively.

All of the CASSCF calculations required for the present work were primarily carried out using Gaussian 03 [56] but, purely for our convenience, the same wavefunctions were also obtained using MOLPRO [57,58]. For reasons that we have explained, it was important to use the ground-state geometries for all of these calculations. We note in passing that accurate excited-state geometry optimizations of antiaromatic states would require methods such as CASPT2, given that those based on a closed-shell reference do not describe correctly the biradical character. Such studies are outside the scope of the present work but may be considered when CASPT2 analytical gradients and Hessians become widely available, making the optimization and characterization of excited-state local minima and saddle points very much faster and more reliable.

### 2.2. Multicentre Bond Indices

Such indices were originally introduced [59,60] as mono-, bi- tri- and generally *k*-centre contributions resulting at the closed-shell SCF or Kohn–Sham level of theory from the identity (1)

(1) $$Tr{\left(PS\right)}^{k}=2^{\left(k-1\right)}N=\underset{A}{\sum }\Delta_{A}^{\left(k\right)}+\underset{A<B}{\sum }\Delta_{AB}^{\left(k\right)}+\underset{A<B<C}{\sum }\Delta_{ABC}^{\left(k\right)}+\ldots \underset{A<B<C\ldots K}{\sum }\Delta_{ABC\ldots K}^{\left(k\right)}$$

in which *P* and *S* denote the charge-density and overlap matrices, respectively, and *N* is the number of electrons. The usefulness of these indices for structural elucidations arises from the interesting nontrivial finding that their values mimic sensitively the presence and/or absence of bonding interactions between individual atoms in a molecule. Thus, for example, in the case of molecules that are well described by the familiar classical Lewis model of localized two-centre two-electron bonds, the corresponding 2-centre bond indices, which coincide in this case with the well-known Wiberg–Mayer indices [61,62], attain non-negligible values only between classically bonded atoms while the corresponding values of the indices for pairs of classically nonbonded atoms are negligible. Such indices are also very useful for molecules whose descriptions transcends the classical Lewis model by involving instead bonding interactions that are delocalized over more than two atomic centres. The indices retain the ability in such cases to detect and reveal just those atomic fragments engaged in the delocalized bonding, whereas the values for the remaining fragments are very small.

In various earlier papers the definition was modified [29,63,64] and the indices were instead normalized to *N*, as shown in Equation (2).

(2) $$\frac{1}{2^{\left(k-1\right)}}Tr{\left(PS\right)}^{k}=N=\underset{A}{\sum }\Delta_{A}^{\left(k\right)}+\underset{A<B}{\sum }\Delta_{AB}^{\left(k\right)}+\underset{A<B<C}{\sum }\Delta_{ABC}^{\left(k\right)}+\ldots \underset{A<B<C\ldots K}{\sum }\Delta_{ABC\ldots K}^{\left(k\right)}$$

A slight disadvantage of this alternative definition is that the resulting values of the indices tend to decrease rather dramatically with increasing *k*. We have thus chosen for the present work to use the original definition (Equation (1)) in which the normalization sum is $Tr{\left(PS\right)}^{k}$ (which is equal to $2^{k-1}N$ at the closed-shell SCF level). Otherwise, even relevant indices would be rather small for *k* > 3. An obvious alternative to the different values returned by Equations (1) and (2) would be to quote instead the proportion of the quantity being “partitioned”.

In the case of *k*-centre indices the general definition (Equation (1)) leads to Equation (3)

(3) $$MCI_{ABC\ldots K}=\Delta_{ABC\ldots K}^{\left(k\right)}=\underset{i}{\sum }\Gamma_{i}\left[\overset{A}{\underset{\mu }{\sum }}\overset{B}{\underset{\nu }{\sum }}\overset{C}{\underset{\lambda }{\sum }}\ldots \overset{K}{\underset{\tau }{\sum }}{\left(PS\right)}_{\mu \nu }{\left(PS\right)}_{\nu \lambda }{\left(PS\right)}_{\lambda \sigma }\ldots {\left(PS\right)}_{\tau \mu }\right]$$

where the permutation operator $\Gamma_{i}$ ensures that the index includes all of the terms that correspond to different permutations of atomic labels.

The above general formula can also be straightforwardly extended beyond the scope of the Hartree-Fock approximation. The formula remains formally the same, except of course that the idempotent charge-density matrix is replaced by the corresponding correlated first-order density matrix [65,66]. The normalization sum $Tr{\left(PS\right)}^{k}$ is of course no longer straightforwardly linked to the total number of electrons, as in the case of the closed-shell SCF approximation. The above definitions of multicentre indices that are based on a Mulliken-like partitioning can easily be generalized to the framework of QTAIM [67] analysis [30,68], such that Equation (4) is transformed to:

(4) $$MCI_{ABC\ldots K}=\underset{i}{\sum }\Gamma_{i}\left[\underset{\alpha }{\sum }\underset{\beta }{\sum }\underset{\gamma }{\sum }\ldots \ldots \underset{\tau }{\sum }\eta_{\alpha }\eta_{\beta }\eta_{\gamma }\ldots \eta_{\tau }\langle \alpha |\beta \rangle_{A}\langle \beta |\gamma \rangle_{B}\langle \gamma |\delta \rangle_{C}\ldots \langle \tau |\alpha \rangle_{K}\right]$$

Here the symbol $\langle \lambda |\sigma \rangle_{X}$ denotes the domain-condensed overlap of natural orbitals λ and σ (i.e., integration over the QTAIM atomic domain of atom X), $\eta_{\lambda }$ denotes the occupation number of λ, and the summations again run over all permutations of atomic labels.

Instead of using orbitals it is of course possible to calculate indices separately for *α* and *β* spin-orbitals. Such an approach was reported in earlier extensions of multicentre indices to open-shell systems [69]. The total index is of course then the sum of the corresponding *α* and *β* contributions. We note that a recent study dealing with the application of multicentre bond indices to the excited-state aromaticity of benzene and cyclobutadiene [47] used natural spin-orbitals (NSOs) instead of natural orbitals (NOs), even for singlet states. The multicentre indices (MCIs) reported in the present study were calculated with our own programs using the QTAIM approach [67], with the required domain-condensed overlaps generated using the AIMAll program [70].

Using NSO rather than NO expansions is of course straightforward for systems with nonzero spin because one may use combinations of the charge-density and spin-density matrices to generate the different NSO expansions for the *α* and *β* one-electron densities. On the other hand, the spin-density matrix is null for singlet states and so the NSO expansions of the *α* and *β* one-electron densities must coincide. Given that the NO occupations are split equally between the *α* and *β* NSOs we may refer to this as the “half-electron scheme”. Because the *α* and *β* NSOs are the same, and coincide with the NOs, the total *α* and *β k*-centre multicentre indices calculated using this scheme are straightforwardly related to those calculated from the NO occupations (Equation (4)) via the trivial scaling factor ${\left(\frac{1}{2}\right)}^{k-1}$.

A potential problem for singlet states can easily be appreciated by noticing that *α* and *β* NSO occupations do not distinguish between the combination of “singlet diradical” determinants $\varphi \bar{\psi }$ and $\bar{\varphi }\psi$ and the combination of “closed shell” determinants $\varphi \bar{\varphi }$ and $\psi \bar{\psi }$. As a consequence, the resulting multicentre indices do not take explicit account of which states have a high degree of diradical character and which of them do not. The degree of diradical character could of course be an important feature for considerations of aromaticity. As such the “half-electron” approach, although formally the correct one, might appear to be slightly questionable when considering, for example, an inherently diradical species such as the singlet ground state of square cyclobutadiene. As was demonstrated in the seminal study by Salem and Rowland [71], the diradicals represented in the simplest model by two degenerate orbitals occupied by two electrons form four electronic states, namely two biradical states (singlet and triplet) and two zwitterionic singlet states:

(5) $$S_{1},T_{1}=\frac{1}{\sqrt{2}}\left(\left|\phi \bar{\psi }\right|\pm \left|\psi \bar{\varphi }\right|\right)\;Z_{1}\pm Z_{2}=\frac{1}{\sqrt{2}}\left(\left|\varphi \bar{\varphi }\right|\pm \left|\psi \bar{\psi }\right|\right)$$

Although all of these states differ at the level of the pair density, and thus also of the energy, all of the spinless one-electron densities coincide:

(6) $$\rho \left(1\right)=\varphi^{2}\left(1\right)+\psi^{2}\left(1\right)$$

Such observations made it seem attractive to consider taking explicit account of singlet diradical character by means of artificial modifications of the *α* and *β* NSO occupations. In the simple case of the singlet ground state of square cyclobutadiene, which features two singly occupied orbitals, we could for example consider that the first of them is pure *α* and the other one is pure *β* spin. We use the label “diradical scheme” for this somewhat artificial approach in which the *α* and *β* densities are now allowed to be different, albeit they still add to the correct total. In actual practice we did unfortunately find that manipulations of this sort were far from satisfactory. There were particular complications and uncertainties for cases such as states of benzene which feature two pairs of degenerate orbitals (each corresponding of course to one of the *E* irreducible representations in *D*_6*h*_ symmetry). We were also concerned that some invariances to orbital rotations might be lost and we noticed that using analogous manipulations for triplet states resulted in “artificial” NSO occupations that bear no obvious resemblance to the actual ones. As a consequence, we reluctantly mostly abandoned this diradical scheme and so we focus here on our results that were obtained for the singlet and triplet states with the actual NSO occupation numbers. Nonetheless, because of this inability of the first-order density matrix to reflect important features that are present in the wavefunction and the pair density, we considered it useful to take into account in our considerations also the eventual manifestation in the wavefunction of diradical character, given that it could be very important in the evaluation of the degree of aromaticity. For this purpose and in order to provide additional insights into the nature of the individual excited states of the molecules studied we also quantify the contributions to the occupation numbers that arise from diradical character. (Note that, instead of using the actual NSO occupations for the singlet states, we could have used Equation (4) with the NO occupations, rescaling the resulting 4- and 6-centre indices by 18 and 132, respectively).

### 2.3. Similarity of Excited States

As an auxiliary tool to assess the aromaticity and/or antiaromaticity of individual excited states we also used a molecular similarity index that is based on the number of overlapping electrons (NOEL), as was introduced some time ago by Cioslowski [72]. In essence, the index of similarity between two molecules *A* and *B* is defined in terms of the first-order density matrices of the corresponding molecules as

(7) $$d_{AB}=\int^{\;}{\left|\Gamma^{A}\left(x,x^{\prime }\right)-\Gamma^{B}\left(x,x^{\prime }\right)\right|}^{2}\;dx\;dx^{\prime }$$

where the density matrices $\Gamma^{X}$ are conveniently represented by NSO expansions. The smaller the values of $d_{AB}$, the more similar are the first-order densities of the molecules *A* and *B*.

NOEL-based comparisons of systems with different geometries would involve also the optimization of the mutual positions of the two molecules, so as to maximize the similarity. In the present work, however, the comparisons of the different states of a given molecules are much more straightforward because of our decision to consider vertical excitations, i.e., fixed geometries. The above general formula then reduces to

(8) $$\begin{array}{cc} d_{AB}=d_{AB}^{\alpha }+d_{AB}^{\beta }= & \\ & Tr\left(P_{A}^{\alpha }S\right)\left(P_{A}^{\alpha }S\right)+Tr\left(P_{B}^{\alpha }S\right)\left(P_{B}^{\alpha }S\right)-2Tr\left(P_{A}^{\alpha }S\right)\left(P_{B}^{\alpha }S\right)+\\ & Tr\left(P_{A}^{\beta }S\right)\left(P_{A}^{\beta }S\right)+Tr\left(P_{B}^{\beta }S\right)\left(P_{B}^{\alpha \beta }S\right)-2Tr\left(P_{A}^{\beta }S\right)\left(P_{B}^{\beta }S\right) \end{array}$$

where $P_{X}^{\alpha }$ and $P_{X}^{\beta }$ denote the *α* and *β* one-electron density matrices, respectively, for a particular electronic state of a given molecule and *S* is the overlap matrix.

## 3. Results and Discussion

As our primary tool for the evaluation of excited-state aromaticity we used the multicentre bond indices whose calculation requires knowledge of the first-order density matrix provided in a quantum chemical calculation via natural orbitals and their occupation numbers. In view of the potential problems mentioned in the previous section, the use of quantities based on the first-order density matrix might not always be a completely satisfactory approach: This matrix is not able to reflect all of the features of a more complicated wavefunction and, in certain cases, the features not carried over could be of crucial importance. The relevance of this concern can be illustrated using simple considerations applied to wavefunctions exhibiting diradical character which are often encountered when describing excited electronic states.

It is of course entirely straightforward to construct an expansion of a CASSCF wavefunction in terms of determinants built from NOs so as to reproduce the already known NO occupation numbers. Then we can determine also the net contributions arising from determinants in which a particular NO is singly occupied. Especially for singlet states, the results provide a useful quantitative measure of the extent of diradical character. In most cases, sufficient qualitative information can be obtained just by examining the compositions of the most important determinants in the expansion and, as shown below, doing so is essential when evaluating the reliability of multicentre indices and NOEL-based similarity values as aromaticity criteria.

The need for a more detailed analysis of the nature of each individual excited state is highlighted by the observation that states of very different character can have fairly similar patterns of NO occupation numbers, as can be seen in Table 1, Table 2 and Table 3. Such similarities are displayed, for example, by the S_1_ and S_2_ states of benzene, as well as by all three singlet states of square cyclobutadiene that we examined. The absence of pronounced differences between the patterns of NO occupation numbers is a cause for concern because it is not clear how the multicentre indices, as well as the NOEL-based $d_{AB}$ values, will be able to distinguish properly between such electronic states unless the shapes of the NOs change sufficiently between states.

The net contributions from all determinants in which a particular NO is singly occupied to the wavefunctions for the various electronic states of benzene, square cyclobutadiene and disulfur dinitride are shown in Table 1, Table 2 and Table 3. Clearly, both the S_1_ state and, of course, the T_1_ state of benzene exhibit significant levels of diradical character, unlike the S_0_ and S_2_ states (see Table 1). In the case of square cyclobutadiene (Table 2) the states with significant levels of diradical character are S_0_ and again, of course, T_1_, whereas there only minor traces of such character in the S_1_ and S_2_ states which appear to be zwitterionic [71]. Moving on to S_2_N_2_, we can see from Table 3 that that it is only the S_0_ ground state that has slight diradical character, whereas there is strong diradical character in both of the excited states. Inspection of the symmetries of the NOs for the electronic states of this molecule reveals that whereas the dominant diradical character in T_1_ comes from two unpaired π electrons, much as in the corresponding states of C_6_H_6_ and C_4_H_4_, that in the S_1_ state is associated with the coupling of an unpaired σ electron to an unpaired π electron. Therefore, we can expect that the NOEL-based similarity index for S_2_N_2_ will show significant differences between the valence σ system of the S_1_ state and those of the S_0_ and T_1_ states.

The values of multicentre QTAIM indices for C_6_H_6_, C_4_H_4_ and S_2_N_2_ calculated using NSOs are shown in Table 4. For singlet states we have used the actual NSO occupations, i.e., the “half-electron” scheme. While inspecting the numbers in this table, it is useful to adopt the largest total 6-centre MCI value of 0.016 for the archetypal example of an aromatic system, the ground state of benzene, as a yardstick for assessing the values of this index for the other states of this molecule. Whereas the value of the index for the first excited singlet state is smaller than its S_0_ counterpart by an order of magnitude, those for the S_2_ and T_1_ states which turn out to very close are, instead, smaller by a factor of about five. It should be mentioned that, in keeping with expectations, the total values of the indices are dominated by their π-only components for all of the states included in Table 4. The situation in the case of S_2_N_2_ is similar to that in C_6_H_6_: Once again, the largest value of the 4-centre MCI is that for the ground state, whereas the excited-state indices are considerably smaller. The 4-centre MCIs for the electronic states of square C_4_H_4_ follow a different pattern: The largest value corresponds to the lowest triplet state T_1_, whereas the indices for the three singlet states are considerably smaller.

The changes in the values of the MCIs in Table 4 between S_0_ and T_1_ states are fully consistent with Baird’s original rule [42] which addresses aromaticity reversals involving the singlet ground and lowest triplet electronic states only. However, when singlet states come into play, there are some notable discrepancies from the behaviour expected on the basis of the results from previous studies which discuss in detail ground- and excited-state magnetic properties, including several types of NICS [44,45,52]. Let us start with benzene. The S_1_ state of C_6_H_6_ is correctly classified as antiaromatic, with S_1_ being more antiaromatic than T_1_ which is in line with predictions based on magnetic properties [44,45] and several multicentre and delocalization indices [47]. However, S_2_ in C_6_H_6_ is predicted to be just as antiaromatic as T_1_ whereas the magnetic properties of this state, calculated at the same geometry and level of theory, strongly suggest that it is even more aromatic than the ground state S_0_ [44]. Incidentally, S_2_ in C_6_H_6_ was classified as more antiaromatic than S_1_ in [47] but this was due to analysing the doubly-degenerate S_4_ rather than S_2_ (for details, see [44]). We continue our analysis with square C_4_H_4_. According to the 4-centre MCI values, S_1_ is the most antiaromatic state of this molecule whereas the isotropic shielding isosurface for this state and other magnetic properties, calculated at the same geometry and level of theory, show clearly that it is aromatic [44]. S_2_ is predicted to be less antiaromatic than S_0_ which is in agreement with the results of magnetic property calculations [44,45]. Finally, according to the respective 4-centre MCI values, in S_2_N_2_ S_1_ is less antiaromatic than T_1_ whereas magnetic property calculations suggest the opposite ordering for these two states [52]. One further observation is that the values of the 6-centre MCI for benzene are, for most states, of smaller magnitude than the corresponding 4-centre MCIs for square cyclobutadiene and disulfur dinitride, which precludes comparisons between MCIs for different rings.

The discrepancies between the current MCI-based assessments of the aromaticities of singlet excited states and those coming from calculations of various magnetic second-order response properties [44,45] underline our concerns as to whether first-order density-based indices are capable of reflecting more subtle effects whose description relies on the more detailed information inherent to wavefunctions or pair densities for particular electronic states.

Similar concerns are associated with comparisons utilizing the NOEL-based quantity *d_AB_*. This quantity was originally designed [72] as a quantitative measure of the similarity between the electron densities of different molecules but, in this study, we use it to investigate the similarity between the electron densities of different excited states of one molecule. The values of the NOEL-based similarity index $d_{AB}$ for the low-lying electronic states of benzene, square cyclobutadiene and disulfur dinitride, calculated using NSOs, are summarized in Table 5, Table 6 and Table 7. These results demonstrate clearly, in keeping with expectations, that the total $d_{AB}$ indices for C_6_H_6_ and C_4_H_4_ are, in all cases, dominated by their π-only components. In the case of S_2_N_2_, $d_{AB}$ indices involving the S_1_ state show large σ contributions due to the composition of the wavefunction for this state (see above).

The data in Table 5 suggest some similarity between the S_0_ and S_2_ states of benzene, in line with the expected aromaticity of S_2_ [44], as well as very little similarity between the S_0_ and T_1_ states, in agreement with Baird’s rule. However, the surprisingly high level of similarity between the S_1_ and S_2_ states which have been classified as antiaromatic and aromatic, respectively [44], is very much out of line with the rather different magnetic properties of these states. Somewhat surprising are also the comparable levels of similarity between the S_1_ and T_1_ states, both of which are supposed to be antiaromatic, and the S_2_ and T_1_ states, which are supposed to be aromatic and antiaromatic, respectively [44].

Other similarity assessments of questionable utility can be found amongst the data for square cyclobutadiene that are presented in Table 6, starting with the high level of similarity between the S_0_ and S_1_ states which is unexpected, in view of the predicted aromaticity reversal between these states [44]. Both S_1_ and T_1_ are expected to be aromatic [44], but the level of similarity between these states is comparable to that between S_2_ and T_1_, which are expected to be antiaromatic and aromatic, respectively.

The $d_{AB}$ indices are doing a better job in the case of S_2_N_2_ (see Table 7): S_0_ and S_1_ are quite dissimilar, and so are S_0_ and T_1_, as expected for comparisons between wavefunctions corresponding to aromatic and antiaromatic states. S_1_ and T_1_ come out as very dissimilar which is not unrealistic, as these states have been predicted to show very different levels of antiaromaticity [52]. As has been mentioned, the σ-only valence contributions to $d_{AB}$ are large in all comparisons involving the first singlet excited state, due to the composition of the S_1_ wavefunction (see above).

We have shown that the multicentre indices (MCIs) examined in this work perform well for aromaticity reversals involving the singlet ground and lowest triplet electronic states which are covered by Baird’s original rule [42]. Our attempts to apply these indices to aromaticity reversals involving singlet excited states were less satisfactory. While this may seem disappointing, since aromaticity/antiaromaticity switching can be predicted even using simple topological resonance energies [43], it should be emphasized that TRE-based studies do not distinguish between singlet and triplet excited states, and all MCI problems arise when dealing with singlet excited states. When dealing with singlet ground states, 6-centre indices have been found to correlate very well with the energetic stabilization resulting from cyclic delocalization in individual benzene rings in polycyclic aromatic hydrocarbons [29,40]; multicentre indices have also been reported as a reliable measure of aromaticity in all-metal clusters [73].

One potential source of the problems experienced when trying to apply multicentre indices to singlet excited states can be associated with the reasons behind the very good performance of MCIs for the ground states of polycyclic aromatic hydrocarbons. The correlation between MCIs and energetic stabilization stems from Coulson’s integral formula [74] which describes quantitatively the extent of energetic stabilization/destabilization associated with cyclic conjugation. However, this formula can be applied only to the ground states of conjugated hydrocarbons and, as there is no equivalent formula for excited states, there is also no straightforward way of measuring the energetic effects resulting from cyclic conjugation in other than ground states (TRE is applicable only to the lowest excited state). The absence of an energy-based justification of excited-state MCIs may have adverse impact on their performance in comparison to ground-state MCIs. On the other hand, cyclic conjugation in excited states can be thought to induce excited-state ring currents and the nature of these currents (paratropic vs diatropic) is decisive for excited aromaticity. These ring currents can be integrated using the Bio-Savart law (as shown, for example, in [75]), producing excited-state magnetic shielding tensors such as those calculated and analysed in [44,45,46,52] which explains why the magnetic properties of excited states provide reliable measures of excited-state aromaticity.

In addition to these somewhat qualitative arguments, more detailed theoretical considerations can be used to identify additional factors affecting the performance of the multicentre indices for singlet excited states. For this purpose, it is useful to refer again to the paper by Salem and Rowland [71] dealing with the electronic structure of diradicals. As we mentioned previously, although all four biradical and zwitterionic states for the simple two-orbital model (see Equation (5)) differ in energy (and, consequently, in wavefunction and in pair density), their one-electron densities are exactly the same. One straightforward implication is that the first-order density matrices for different electronic states of real systems could omit important details, the absence of which would result in multicentre indices giving misleading information about the extent of similarity between these states. Although the discussion in [71] is focused on inherently diradical species, similar problems, arising from details not available within the first-order density matrix, are apparently more general since, as demonstrated in this study, partial diradical character is evident even in the excited-state wavefunctions of a paradigmatic molecule such as benzene. On the other hand, the undeniable usefulness of multicentre indices as a measure of ground-state aromaticity [29,30,32,40,73] can be attributed to the fact that, at the closed-shell SCF and Kohn–Sham levels of theory, the first-order density matrix determines all higher-order densities so that energy-related quantities are described correctly.

## Figures and Tables

**Table 1:** Active-space NO occupation numbers for the S_0_, S_1_, S_2_ and T_1_ states of C_6_H_6_ and active-space NSO occupation numbers for the T_1_ state (all in descending order). Values in brackets show the net contributions from all determinants in which a particular NO is singly occupied.

	S_0_	S_1_	S_2_	T_1_	T_1_	
					*α*	*β*
*η*_1_	1.961 (0.028)	1.863 (0.107)	1.952 (0.039)	1.910 (0.078)	0.986	0.924
*η*_2_	1.902 (0.046)	1.445 (0.500)	1.494 (0.051)	1.464 (0.487)	0.966	0.498
*η*_3_	1.902 (0.046)	1.445 (0.500)	1.494 (0.051)	1.464 (0.487)	0.966	0.498
*η*_4_	0.100 (0.046)	0.569 (0.500)	0.524 (0.051)	0.536 (0.487)	0.503	0.034
*η*_5_	0.100 (0.046)	0.569 (0.500)	0.524 (0.051)	0.536 (0.487)	0.503	0.034
*η*_6_	0.036 (0.028)	0.109 (0.087)	0.012 (0.007)	0.090 (0.079)	0.077	0.013

**Table 2:** Active-space NO occupation numbers for the S_0_, S_1_, S_2_ and T_1_ states of square C_4_H_4_ and active-space NSO occupation numbers for the T_1_ state (all in descending order). Values in brackets show the net contributions from all determinants in which a particular NO is singly occupied.

	S_0_	S_1_	S_2_	T_1_	T_1_	
					*α*	*β*
*η*_1_	1.905 (0.073)	1.835 (0.031)	1.994 (0.001)	1.914 (0.072)	0.993	0.921
*η*_2_	1.000 (1.000)	1.005 (0.000)	1.000 (0.000)	1.000 (1.000)	0.964	0.036
*η*_3_	1.000 (1.000)	1.005 (0.000)	1.000 (0.000)	1.000 (1.000)	0.964	0.036
*η*_4_	0.095 (0.073)	0.155 (0.031)	0.006 (0.001)	0.086 (0.072)	0.079	0.007

**Table 3:** Active-space NO occupation numbers for the S_0_, S_1_ and T_1_ states of S_2_N_2_ and active-space NSO occupation numbers for the T_1_ state (all in descending order). Values in brackets show the net contributions from all determinants in which a particular NO is singly occupied.

	S_0_	S_1_	T_1_	T_1_	
				*α*	*β*
*η*_1_	1.997 (0.002)	1.997 (0.002)	1.998 (0.002)	0.999	0.999
*η*_2_	1.997 (0.002)	1.996 (0.003)	1.997 (0.002)	0.999	0.998
*η*_3_	1.996 (0.003)	1.994 (0.005)	1.995 (0.004)	0.999	0.998
*η*_4_	1.991 (0.005)	1.990 (0.007)	1.994 (0.006)	0.998	0.996
*η*_5_	1.986 (0.012)	1.984 (0.013)	1.993 (0.005)	0.998	0.994
*η*_6_	1.981 (0.016)	1.968 (0.025)	1.983 (0.016)	0.997	0.991
*η*_7_	1.980 (0.010)	1.965 (0.028)	1.982 (0.015)	0.997	0.984
*η*_8_	1.964 (0.028)	1.960 (0.031)	1.967 (0.027)	0.996	0.983
*η*_9_	1.962 (0.030)	1.960 (0.035)	1.965 (0.024)	0.991	0.983
*η*_10_	1.960 (0.030)	1.911 (0.034)	1.965 (0.025)	0.984	0.982
*η*_11_	1.908 (0.030)	1.009 (0.979)	1.014 (0.984)	0.983	0.017
*η*_12_	0.126 (0.047)	0.995 (0.970)	1.008 (0.986)	0.983	0.017
*η*_13_	0.041 (0.031)	0.126 (0.060)	0.037 (0.026)	0.019	0.016
*η*_14_	0.040 (0.029)	0.058 (0.045)	0.037 (0.026)	0.019	0.016
*η*_15_	0.038 (0.030)	0.044 (0.032)	0.035 (0.026)	0.019	0.014
*η*_16_	0.033 (0.024)	0.043 (0.031)	0.031 (0.022)	0.017	0.012

**Table 4:** 6-centre MCIs for the S_0_, S_1_, S_2_ and T_1_ states of C_6_H_6_, 4-centre MCIs for the S_0_, S_1_, S_2_ and T_1_ states of square C_4_H_4_ and 4-centre MCIs for the S_0_, S_1_ and T_1_ states of S_2_N_2_. QTAIM/6-311++G(2d,2p) for C_6_H_6_ and C_4_H_4_; QTAIM/cc-pVTZ for S_2_N_2_. Values in brackets show the π-only (C_6_H_6_ and C_4_H_4_) and π-only valence (S_2_N_2_) components of the total index.

	C_6_H_6_	C_4_H_4_	S_2_N_2_
S_0_	0.0160 (0.0159)	0.0360 (0.0336)	0.0525 (0.0504)
S_1_	0.0016 (0.0014)	0.0263 (0.0240)	0.0128 (0.0164)
S_2_	0.0029 (0.0027)	0.0477 (0.0454)	
T_1_	0.0030 (0.0029)	0.0774 (0.0749)	−0.0045 (−0.0069)

**Table 5:** Similarity indices $d_{AB}$ calculated at the CASSCF(6,6)/6-311++G(2d,2p) level for low-lying electronic states of C_6_H_6_. Values in brackets are π-only contributions to the total index.

State	S_1_	S_2_	T_1_
**S_0_**	0.4402 (0.4396)	0.3639 (0.3612)	0.8323 (0.8319)
**S_1_**		0.0453 (0.0433)	0.4465 (0.4464)
**S_2_**			0.4866 (0.4842)

**Table 6:** Similarity indices $d_{AB}$ calculated at the CASSCF(4,4)/6-311++G(2d,2p) level for low-lying electronic states of square C_4_H_4_. Values in brackets are π-only contributions to the total index.

State	S_1_	S_2_	T_1_
**S_0_**	0.0121 (0.0117)	0.0557 (0.0532)	0.8672 (0.8672)
**S_1_**		0.0417 (0.0408)	0.8754 (0.8752)
**S_2_**			0.9086 (0.9067)

**Table 7:** Similarity indices $d_{AB}$ calculated at the CASSCF(22,16)/cc-pVTZ level for low-lying electronic states of S_2_N_2_. Values in brackets are (π-only, σ-only) valence contributions to the total index.

State	S_1_	T_1_
**S_0_**	0.9596 (0.4796, 0.4799)	1.8061 (1.7896, 0.0166)
**S_1_**		2.8024 (2.2926, 0.5098)

## References

[1] Hückel E. (1931). Quantentheoretische Beiträge zum Benzolproblem I. Die Elektronenkonfiguration des Benzol und verwandter Verbindungen. Z. Phys..

[2] Hückel E. (1932). Beiträge zum Problem der aromatischen und ungesättigten Verbindungen. Z. Phys..

[3] Streitwieser A. (1961). Molecular Orbital Theory for Organic Chemists.

[4] Schaad L.J., Hess B.A. (1971). Hückel molecular orbital π resonance energies. A new approach. J. Am. Chem. Soc..

[5] Dewar M.J.S., Gleicher G.J. (1965). Ground states of conjugated molecules. II. Allowance for molecular geometry. J. Am. Chem. Soc..

[6] Baird N.C. (1971). Dewar resonance energy. J. Chem. Educ..

[7] Schaad L.J., Hess B.A. (2001). Dewar resonance energy. Chem. Rev..

[8] Aihara J. (1976). A new definition of Dewar-type resonance energies. J. Am. Chem. Soc..

[9] Gutman I., Milun M., Trinajstič N. (1977). Graph theory and molecular orbitals. 19. Nonparametric resonance energies for arbitrary conjugated hydrocarbons. J. Am. Chem. Soc..

[10] Kuwajima S. (1984). Valence bond theory of aromaticity. J. Am. Chem. Soc..

[11] Pauling L. (1936). The diamagnetic anisotropy of aromatic molecules. J. Chem. Phys..

[12] London F. (1937). Théorie quantique des courants interatomiques dans les combinaisons aromatiques. J. Phys. Radium.

[13] Pople J.A. (1956). Proton magnetic resonance of hydrocarbons. J. Chem. Phys..

[14] Pople J.A., Untch K.G. (1966). Induced paramagnetic ring currents. J. Am. Chem. Soc..

[15] Jug K. (1983). A bond order approach to ring current and aromaticity. J. Org. Chem..

[16] Gomes J.A.N.F., Mallion R. (2001). Aromaticity and ring currents. Chem. Rev..

[17] Fowler P.J., Steiner E., Havenith R.W.A., Jenneskens L.W. (2004). Current density, chemical shifts and aromaticity. Magn. Reson. Chem..

[18] Schleyer P.V.R., Maerker C., Dransfeld A., Jiao H., Van Eikema Hommes N.J.R. (1966). Nucleus-independent chemical shifts: A simple and efficient aromaticity probe. J. Am. Chem. Soc..

[19] Aihara J. (2006). Circuit Resonance Energy. A key quantity that links energetic and magnetic criteria of aromaticity. J. Am. Chem. Soc..

[20] Evans M.G., Warhurst E. (1938). The activation energy of diene association reactions. Trans. Faraday Soc..

[21] Dewar M.J.S. (1971). Aromaticity and pericyclic reactions. Angew. Chem. Int. Ed. Engl..

[22] Van der Hart W.J., Mulder J.C.C., Oosterhof L.J. (1972). Extended valence bond theory, aromaticity and the Woodward-Hoffmann rules. J. Am. Chem. Soc..

[23] Aihara J. (1978). Aromaticity-based theory of pericyclic reactions. Bull. Chem. Soc. Jpn..

[24] Kruszewski J., Krygowski T.M. (1972). Definition of aromaticity basing on the harmonic oscillator model. Tetrahedron Lett..

[25] Krygowski T.M., Cyrański M.K. (2001). Structural aspects of aromaticity. Chem. Rev..

[26] Kleinpeter E., Koch A. (2012). Antiaromaticity proved by anisotropic effect in ^1^H NMR spectra. J. Phys. Chem. A.

[27] Karadakov P.B., Horner K.E. (2013). Magnetic shielding in and around benzene and cyclobutadiene: A source of information about aromaticity, antiaromaticity and chemical bonding. J. Phys. Chem. A.

[28] Giambiagi M., De Giambiagi M.S., Dos Santos Silva C.D., De Fuguereido A.P. (2000). Multicenter bond indices as a measure of aromaticity. Phys. Chem. Chem. Phys..

[29] Bultinck P., Ponec R., Van Damme S. (2005). Multicenter bond indices as a new measure of aromaticity of polycyclic aromatic hydrocarbons. J. Phys. Org. Chem..

[30] Mandado M., Gonzáles M.J., Mosquera R.A. (2007). QTAIM *n*-center delocalization indices as descriptors of aromaticity in mono and poly heterocycles. J. Comp. Chem..

[31] Polansky O.E., Derflinger G. (1967). Zur Clar’schen Theorie Lokaler Benzoider Gebiete in Kondensierten Aromaten. Int. J. Quant. Chem..

[32] Bultinck P., Ponec R., Gallegos A., Fijas S., Van Damme S., Carbó-Dorca R. (2006). Generalized polansky index as an aromaticity measure in polycyclic aromatic hydrocarbons. Croat. Chem. Acta.

[33] Matito E., Duran M., Solà M. (2005). The aromatic fluctuation index (FLU): A new aromaticity index based on electron delocalization. J. Chem. Phys..

[34] Jug K., Köster A.M. (1991). Aromaticity as a multi-dimensional phenomenon. J. Phys. Org. Chem..

[35] Katritzky A.R., Karelson M., Sild S., Krygowski T.M., Jug K. (1998). Aromaticity as a quantitative concept. 7. aromaticity reaffirmed as a multidimensional characteristic. J. Org. Chem..

[36] Katritzky A.R., Baczynski P., Musumarra G., Pisani D., Szafran M. (1989). Aromaticity as a quantitative concept. A statistical demonstration of the orthogonality of classical and magnetic aromaticity of five- and six-membered heterocycles. J. Am. Chem. Soc..

[37] Fijas S., Fowler P.W., Delgado J.L., Hahn U., Bultinck P. (2008). Correlation of delocalization indices and current-density maps in polycyclic aromatic hydrocarbons. Chem. Eur. J..

[38] Cieselski A., Krygowski T.M., Cyrański M.K., Dobrowolski M.A., Aihara J. (2009). Graph-topological approach to magnetic properties of benzenoid hydrocarbons. Phys. Chem. Chem. Phys..

[39] Fijas S., Van Damme S., Bultinck P. (2008). Multidimensionality of delocalization indices and nucleus independent chemical shifts in polycyclic aromatic hydrocarbons. J. Comp. Chem..

[40] Ponec R., Fijas S., Van Damme S., Bultinck P., Gurman I., Stanković S. (2009). The close relation between cyclic delocalization, energy effects of cycles and aromaticity. Coll. Czech. Chem. Commun.

[41] Mandado M. (2010). Theoretical basis for the correlation between energetic, magnetic and electron density criteria of aromaticity: Definition of molecular circuit electric conductance. Theor. Chem. Acc..

[42] Baird N.C. (1972). Quantum organic photochemistry. II. Resonance and aromaticity in the lowest ^3^ππ state of cyclic hydrocarbons. J. Am. Chem. Soc..

[43] Ilić P., Sinković B., Trinajstić N. (1980). Topological resonance energies of conjugated structures. Isr. J. Chem..

[44] Karadakov P.B., Hearnshaw P., Horner K.E. (2016). Magnetic shielding, aromaticity, antiaromaticity, and bonding in the low-lying electronic states of benzene and cyclobutadiene. J. Org. Chem..

[45] Karadakov P.B. (2008). Ground and excited-state aromaticity in benzene and cyclobutadiene. J. Phys. Chem. A.

[46] Karadakov P.B. (2008). Aromaticity and antiaromaticity in the low-lying electronic states of cyclooctatetraene. J. Phys. Chem. A.

[47] Feixas F., Vandenbussche J., Bultinck P., Matito E., Solà M. (2011). Electron delocalization and aromaticity in low lying excited states of archetypal organic compounds. Phys. Chem. Chem. Phys..

[48] Ayub R., Papadakis R., Jorner K., Zietz B., Ottoson H. (2017). The cyclopropyl group: An excited state aromaticity indicator?. Chem. Eur. J..

[49] Papadakis R., Ottoson H. (2015). The excited state antiaromatic benzene ring: A molecular Mr. Hyde?. Chem. Soc. Rev..

[50] Ottoson H. (2012). Organic photochemistry: Exciting excited-state aromaticity. Nat. Chem..

[51] Oh J., Sung M.Y., Hong Y., Kim D. (2018). Spectroscopic diagnosis of excited-state aromaticity: Capturing electronic structures and conformations upon aromaticity reversal. Acc. Chem. Res..

[52] Karadakov P.B., Al-Yassiri M.A.M., Cooper D.L. (2018). Magnetic shielding, aromaticity, antiaromaticity and bonding in the low-lying electronic states of S_2_N_2_. Chem. Eur. J..

[53] Cabana A., Bachand J., Giguère J. (1974). The ν_4_ vibration–rotation bands of C_6_H_6_ and C_6_D_6_: The analysis of the bands and the determination of the bond lengths. Can. J. Phys..

[54] Eckert-Maksíc M., Vazdar M., Barbatti M., Lischka H., Maksíc Z.B. (2006). Automerization reaction of cyclobutadiene and its barrier height: An ab initio benchmark multireference average quadratic coupled cluster study. J. Chem. Phys..

[55] Perrin A., Flores Antognini A., Zeng X., Beckers H., Willner H., Rauhut G. (2014). Vibrational spectrum and gas-phase structure of disulfur dinitride (S_2_N_2_). Chem. Eur. J..

[56] Frisch M.J., Trucks G.W., Schlegel H.B., Scuseria G.E., Robb M.A., Cheeseman J.R., Montgomery J.A., Vreven T., Kudin K.N., Burant J.C. (2004). Gaussian 03, Revision A.01.

[57] Werner H.-J., Knowles P.J., Knizia G., Manby F.R., Schütz M. (2012). Molpro: A general-purpose quantum chemistry program package. WIREs Comput. Mol. Sci..

[58] Werner H.-J., Knowles P.J., Knizia G., Manby F.R., Schütz M., Celani P., Györffy W., Kats D., Korona T., Lindh R. MOLPRO, Version 2019.2, A Package of Ab Initio Programs, Cardiff, UK. http://www.molpro.net.

[59] Giambiagi M., De Giambiagi M.S., Mundim K.C. (1990). Definition of a multicentre bond index. Struct. Chem..

[60] Kar T., Sannigrahi A.B. (1990). 3-Center bond index. Chem. Phys. Lett..

[61] Wiberg K.B. (1968). Application of the Pople-Santry-Segal CNDO method to the cyclopropylcarbinyl and cyclobutyl cation and to bicyclobutane. Tetrahedron.

[62] Mayer I. (1986). Bond orders and valences from ab initio wave functions. Int. J. Quantum Chem..

[63] Ponec R., Mayer I. (1997). Investigation of some properties of multicenter bond indices. J. Phys. Chem. A.

[64] Ponec R., Yuzhakov G. (2004). Multicenter bonding in organic chemistry. Geometry sensitive 3c–2e bonding in (C…H…C) fragments of organic cations. J. Org. Chem..

[65] Ponec R., Mandado M. (2009). Electron reorganization in allowed and forbidden pericyclic reactions: Multicentre bond indices as a measure of aromaticity and/or antiaromaticity in transition states of pericyclic electrocyclizations. J. Phys. Org. Chem..

[66] Matito E., Solà M., Salvador P., Duran M. (2007). Electron sharing indices at the correlated level. application to aromaticity calculations. Faraday Disc..

[67] Bader R.F.W. (1990). Atoms in Molecules. A Quantum Theory.

[68] Bochicchio R., Ponec R., Torre A., Lain L. (2001). Multicenter bonding within AIM theory. Theor. Chem. Acc..

[69] Ponec R., Torre A., Lain L., Bochicchio R.C. (2000). Multicenter bonding in open-shell systems. A nonlinear population analysis approach. Int. J. Quant. Chem..

[70] Keith T.A. AIMAll (Version 19.10.12). TK Gristmill Software, Overland Park, KS, USA. http://aim.tkgristmill.com.

[71] Salem L., Rowland C. (1972). The electronic properties of diradicals. Angew. Chem. Int. Ed. Engl..

[72] Cioslowski J., Fleischmann E.D. (1991). Assessing molecular similarity from results of ab-initio electronic structure calculations. J. Am. Chem. Soc..

[73] Feixas F., Jimenez-Halla J.O.C., Matito E., Poater J., Solà M. (2010). A test to evaluate the performance of aromaticity descriptors in all-metal and semimetal clusters. an appraisal of electronic and magnetic indicators of aromaticity. J. Chem. Theory. Comp..

[74] Coulson C.A. (1940). On the calculation of the energy in unsaturated hydrocarbon molecules. Proc. Camb. Phil. Soc..

[75] Irons T.J.P., Spence L., David G., Speake B.T., Helgaker T., Teale A.M. (2020). Analyzing magnetically induced currents in molecular systems using current-density-functional theory. J. Phys. Chem. A.

